# Clinical and psychosocial stress factors are associated with decline in physical activity over time in children with juvenile idiopathic arthritis

**DOI:** 10.1186/s12969-021-00584-4

**Published:** 2021-06-29

**Authors:** Liane D. Heale, Kristin M. Houghton, Elham Rezaei, Adam D. G. Baxter-Jones, Susan M. Tupper, Nazeem Muhajarine, Susanne M. Benseler, Gilles Boire, David A. Cabral, Sarah Campillo, Gaëlle Chédeville, Anne-Laure Chetaille, Paul Dancey, Ciaran Duffy, Karen Watanabe Duffy, Janet Ellsworth, Jaime Guzman, Adam M. Huber, Roman Jurencak, Bianca Lang, Ronald M. Laxer, Kimberly Morishita, Kiem G. Oen, Ross E. Petty, Suzanne E. Ramsey, Johannes Roth, Rayfel Schneider, Rosie Scuccimarri, Lynn Spiegel, Elizabeth Stringer, Shirley M. L. Tse, Lori B. Tucker, Stuart E. Turvey, Rae S. M. Yeung, Alan M. Rosenberg, for the BBOP Study Group

**Affiliations:** 1 McMaster Children’s Hospital and McMaster University, Hamilton, Canada; 2 British Columbia Children’s Hospital and University of British Columbia, 4480 Oak Street, Room K4-120, Vancouver, BC V6H 3V4 Canada; 3 Jim Pattison Children’s Hospital and University of Saskatchewan, Saskatoon, Canada; 4 University of Saskatchewan, Saskatoon, Canada; 5 Alberta Children’s Hospital and University of Calgary, Calgary, Canada; 6 Centre Intégré Universitaire de Santé et de Services Sociaux de l’Estrie– Centre Hospitalier Universitaire de Santé (CIUSSS de l’Estrie-CHUS) and University of Sherbrooke, Sherbrooke, Canada; 7 Montreal Children’s Hospital and McGill University, Montreal, Canada; 8 le Centre Hospitalier Universitaire de Quebec, Quebec, Canada; 9 Janeway Children’s Health and Rehabilitation Centre and Memorial University, St. John’s, Canada; 10 Children’s Hospital of Eastern Ontario and University of Ottawa, Ottawa, Canada; 11 Stollery Children’s Hospital and University of Alberta, Edmonton, Canada; 12 IWK Health Centre and Dalhousie University, Halifax, Canada; 13 The Hospital for Sick Children and University of Toronto, Toronto, Canada; 14 The Children’s Hospital of Winnipeg and University of Manitoba, Winnipeg, Canada

**Keywords:** Juvenile arthritis, Physical activity, Psychosocial stress

## Abstract

**Background:**

Physical activity (PA) patterns in children with juvenile idiopathic arthritis (JIA) over time are not well described. The aim of this study was to describe associations of physical activity (PA) with disease activity, function, pain, and psychosocial stress in the 2 years following diagnosis in an inception cohort of children with juvenile idiopathic arthritis (JIA).

**Methods:**

In 82 children with newly diagnosed JIA, PA levels, prospectively determined at enrollment, 12 and 24 months using the Physical Activity Questionnaire for Children (PAQ-C) and Adolescents (PAQ-A) raw scores, were evaluated in relation to disease activity as reflected by arthritis activity (Juvenile Arthritis Disease Activity Score (JADAS-71)), function, pain, and psychosocial stresses using a linear mixed model approach. Results in the JIA cohort were compared to normative Pediatric Bone Mineral Accrual Study data derived from healthy children using z-scores.

**Results:**

At enrollment, PA z-score levels of study participants were lower than those in the normative population (median z-score − 0.356; *p* = 0.005). At enrollment, PA raw scores were negatively associated with the psychosocial domain of the Juvenile Arthritis Quality of Life Questionnaire (*r* = − 0.251; *p* = 0.023). There was a significant decline in PAQ-C/A raw scores from baseline (median and IQR: 2.6, 1.4–3.1) to 24 months (median and IQR: 2.1, 1.4–2.7; *p* = 0.003). The linear mixed-effect model showed that PAQ-C/A raw scores in children with JIA decreased as age, disease duration, and ESR increased. The PAQ-C/A raw scores of the participants was also negatively influenced by an increase in disease activity as measured by the JADAS-71 (*p* <  0.001).

**Conclusion:**

Canadian children with newly diagnosed JIA have lower PA levels than healthy children. The decline in PA levels over time was associated with disease activity and higher disease-specific psychosocial stress.

**Supplementary Information:**

The online version contains supplementary material available at 10.1186/s12969-021-00584-4.

## Background

Children with juvenile idiopathic arthritis (JIA) have been reported to be less active than their healthy peers [1]. However, other reports noted similar JIA physical activity (PA) levels at the time of diagnosis and over a short and longer term follow up when compared to healthy children [2, 3]. These discrepancies could be the result of improved JIA treatment and PA counseling, but could also result from differences in study design, PA measure, populations studied, and length of follow-up. Prospective, longitudinal studies are required to characterize determinants and patterns of PA trajectories in JIA to enable the identification of time dependent effects.

Although PA levels in children and adolescents with JIA have been reported to be negatively associated with disease activity [4–6], disease-dependent factors account for a low percentage of the variance in their PA levels [5]. Adequate disease control does not appear to restore PA levels to that of healthy age-matched controls [4, 6]. Additionally, there is no established relationship between PA levels and measures of functional ability [4–7]. Although pain was highlighted as one barrier to PA in one qualitative study of children with JIA [8], the relationship between PA and pain is complex, and another study had conflicting results [9].

Many non-disease-related factors may influence PA level in children with JIA. In healthy children and adolescents, high socioeconomic status (SES) is associated with higher PA levels [10, 11]. In JIA, low SES is associated with more functional limitations [12]. Further, parental distress, child self-efficacy and social support, are strong predictors of health-related quality of life in newly diagnosed youth with JIA [13], and these could also influence PA. A recent study identified parental support and enjoyment as facilitators and time pressures as barriers for JIA participants in an exercise intervention [14]. A better understanding of the impact of SES and psychosocial factors on PA level could help identify at risk patients who would benefit from additional intervention and support.

To discover the pattern of PA in children with JIA over time and its disease and non-disease determinants, this study aimed to 1) describe the trajectory of PA from diagnosis to 24-month follow up of an inception cohort of children with JIA; 2) compare the PA levels with age- and sex-matched normative data; and 3) examine the association between PA levels and JIA category, disease activity and measures of SES and psychosocial stress. We hypothesized that PA levels at diagnosis would be similar to age- and sex-matched normative data and that ongoing disease activity, low SES and high psychosocial stress would predict a decline in PA levels following diagnosis.

## Materials and methods

### Design

This study was part of a multi-site, longitudinal cohort study of Canadian children with newly diagnosed JIA (BBOP; Biologically-based Outcome Predictors in JIA). BBOP aimed to identify the inter-relationships of biologic, environmental, and lifestyle factors as predictors of childhood arthritis outcomes [15]. We analyzed BBOP data to characterize the trajectory of PA levels in JIA.

Participants had a clinical assessment and completed all study questionnaires at baseline, 12 and 24 months. Where developmentally appropriate, children completed their own self-report questionnaires. Otherwise, they were completed by a parent.

In compliance with the Helsinki Declaration, the study was approved by the Biomedical Research Ethics Board, University of Saskatchewan: #07–86 and by the research ethics boards at each of the other 10 participating sites. Parents and, as appropriate, children provided written informed consent; children provided assent, as applicable.

### BBOP recruitment

Children with a new diagnosis of JIA were enrolled at 11 Canadian pediatric rheumatology centers between March 2008 and January 2011. Enrollment criteria included: (i) consenting participants who met International League of Associations for Rheumatology (ILAR) JIA classification criteria [16] and (ii) were diagnosed within 6 months of symptom onset. As physical activity assessment tools were applicable only for participants aged 6 to 16 years, younger BBOP participants were not included. The cohort comprised participants from each of the seven ILAR JIA subtypes. The aim was to recruit sufficient numbers for each category rather than to strive to achieve a typical JIA subset distribution. To achieve this, only participants with polyarthritis or systemic JIA (the least prevalent subtypes) were eligible during the first 6 months of enrollment after which any JIA subtype was eligible. Participants who were unable to communicate in English or French were excluded from the study.

### Reference population

The reference group for PA measures for this BBOP study analysis were the 154 Canadian children (82 females and 72 males) who participated in the University of Saskatchewan’s Pediatric Bone Mineral Accrual Study (1991–97, entry age 8 to 15 years) [17].

### Measurements

#### Physical activity

Participants completed the Physical Activity Questionnaire (PAQ) at all three study visits [18, 19]. The PAQ is a self-administered, 7-day physical activity recall questionnaire that assesses participation in different physical activities, as well as activity during physical education class, lunch break, recess, after school, in the evenings and on weekends. The Physical Activity Questionnaire for Children (PAQ-C) has been validated in children 5 to 12 years of age [20] and was completed by school age children ≥6 years and ≤ 12 years of age in this study [18]. The Physical Activity Questionnaire for Adolescents (PAQ-A) is recommended for adolescents and was completed by children ≥13 years of age [21]. The first page of the PAQ-A is shown in Supplementary Figure 1. The PAQ-C/A was completed at visits during the school year and through the summer break. Both measures have been validated in healthy children and in children with chronic disease [21, 22].

The PAQ-A and PAQ-C comprise 8 and 9 PA questionnaire items, respectively. Each item is scored between 1 (low PA) and 5 (high PA); a mean score of all items constitutes the overall PAQ score. Questionnaire items 9 (PAQ-A) and 10 (PAQ-C), which ask participants whether anything prevented them from doing normal physical activities, are only used to gauge whether the responses represent the individual’s typical PA levels and are not included in the calculation of the PAQ-C/A score.

#### Clinical assessment

The attending pediatric rheumatologist recorded the total number of joints with active arthritis (0–71 joints) and provided a physician global assessment (PGA) of participant disease activity measured on a 0–10 visual analog scale (VAS) where 0 = no activity and 10 = maximum activity. Participants were asked to complete a parent/patient global assessment of well-being, measured on a 10-cm VAS where 0 = very well and 10 = very poor. If participants required bloodwork as part of their routine clinical care, the erythrocyte sedimentation rate (ESR) was recorded and normalized to a 0–10 scale as outlined by Consolaro et al. [23] The PGA of disease activity, parent/patient global assessment of well-being, active joint count, and ESR were used to calculate the Juvenile Arthritis Disease Activity Score (JADAS) [24]. The JADAS-71 was calculated as the simple sum of the scores of its 4 components, which yields a global score of 0–101.

Participants completed the Child Health Assessment Questionnaire (CHAQ) [25] as a parent-reported measure of their functional impairment. As part of the CHAQ, they were asked to rate their pain in the past week on a 10-cm visual analogue scale (VAS) from 0 (no pain) to 10 (very severe pain) [26, 27].

#### Socioeconomic information

Participants completed a questionnaire at study enrollment that ascertained place of residence (as determined by the Canadian postal code forward sortation area), population of the community in which the participant resided, parental marital status, parental education, parental occupation, and household income.

#### Psychosocial stress

Three self-administered questionnaires were completed at all study visits to assess psychosocial stressors.

The Juvenile Arthritis Quality of Life Questionnaire (JAQQ) measures physical and psychosocial functioning, specifically rating difficulty with functions due to arthritis or its treatment [28]. The JAQQ is a self-administered questionnaire that is applicable to all age groups and JIA subtypes. It consists of 74 items distributed across 4 dimensions. The psychosocial function dimension was used for this study and includes 22 items that are scored in terms of severity from 1 (none of the time) to 7 (all of the time). The mean of the 5 highest scoring items was calculated to give the JAQQ-psychosocial score. Mean scores range from 1 to 7, with 7 indicating the worst function.

The Children’s Hassles Scale measures the frequency and impact of daily hassles, defined as irritating and/or distressing demands that to some degree characterize everyday interactions with the environment [29]. It consists of 25 items scored from 0 (this did not happen) to 3 (yes this happened, and it made you feel very sad). Mean scores range from 0 to 3, with 3 indicating the highest impact of daily hassles.

The Stressful Life Events Checklist (SLEC) measures the occurrence of stressful life events in the prior year [30]. Two versions of the checklist were used: (i) a 40-item checklist for children < 12 years with total scores ranging from 0 (no stressors occurred in the past year) to 40 (all stressors occurred in the past year) and (ii) a 47-item checklist for adolescents ≥12 with total scores ranging from 0 to 47.

Examples of the JAQQ, Hassles Scale and SLEC questionnaire items are shown in Supplementary Table 1.

### Statistical analysis

Statistical analyses were performed with SPSS Statistics Professional, version 23, R, version 3.2.2., and MATLAB R2019a. The data had 21% missing values, which were imputed by Amelia II, R package which imputes missing values employing an expectation maximization algorithm [31, 32]. Age- and sex-specific z-scores for the PAQ-C/A were generated for 8 to 15-year-old participants using normative data from the Saskatchewan Pediatric Bone Mineral Accrual Study [33]. *Mann*–*Whitney U* test was used to examine the differences between girls’ and boys’ PA scores. The Kruskal-Wallis test was used to assess the difference between PA scores of the seven JIA subtypes. The associations between PAQ-C/A and clinical disease activity (JADAS-71), function (CHAQ), SES and measures of psychosocial stress (JAQQ psychosocial domain, SLEC, and Hassles scale) were assessed at baseline using Spearman correlations. A linear mixed model for panel data was used to estimate the association between PA over time with age, sex, and disease activity measures including number of active joints, ESR, c-reactive protein (CRP), and JADAS-71 (fixed effect). The measures of psychosocial stress and function were considered as variables with random effects. The best model was selected using a theoretical likelihood ratio test.

## Results

Eighty-two children (71% female, mean age 12.4 ± 2.9, range 6–16 years) completed the PAQ-C/A at baseline, 12 and 24 months. Descriptive characteristics of the study population at the time of enrollment (baseline) are shown in Table 1.

**Table 1:** Descriptive characteristics of cohort at baseline

	All(*n* = 82) Median (IQR)	Girls(*n* = 58) Median (IQR)	Boys(*n* = 24) Median (IQR)
Participant characteristics			
Age	12.0 (9.0–14.0)	13.0 (10.7–15.0)	9.5 (7.2–12.0)
BMI percentile	60.0 (30.9–84.3)	63.6 (30.9–84.2)	50.0 (30.0–86.5)
PAQ-C/A (scale range 1–5)	2.6 (1.4–3.1)	2.5 (1.3–3.0)	3.0 (1.9–3.9)
JIA subtype			
Oligoarthritis	16	11	5
Polyarthritis RF-	30	23	7
Polyarthritis RF+	8	8	0
Systemic arthritis	11	4	7
Psoriatic arthritis	7	6	1
Enthesitis related arthritis	6	4	2
Undifferentiated arthritis	4	2	2
Disease factors (scale range)			
JADAS-71 (0–101)	15.0 (7.7–20.0)	16.0 (11.7–21.0)	8.5 (5.2–17.0)
CHAQ (0–3)	0.63 (0.25–1.25)	0.63 (0.25–1.25)	0.69 (0.03–1.37)
Pain (CHAQ) (10-cm VAS)	4.0 (2.0–6.0)	4.0 (2.0–6.0)	3.5 (1.0–6.7)
Psychosocial factors (scale range)			
JAQQ (section 3; 1–7)	1.4 (1.1–2.1)	1.4 (1.1–1.9)	1.3 (1.1–2.4)
Hassles scale (0–3)	0.50 (0.24–1.12)	0.46 (0.20–0.92)	0.60 (0.29–1.15)
SLEC (0–47)	10.0 (5.0–15.6)	7.5 (2.5–15.0)	13.7 (7.5–20.0)

*IQR* inter quartile range, *RF* rheumatoid factor, *BMI* body mass index, *PAQ-C/A* physical activity questionnaire for children and adolescents, *JADAS-71* Juvenile Arthritis Disease Activity Score, *CHAQ* Child Health Assessment Questionnaire, *VAS* visual analog scale, *JAQQ* Juvenile Arthritis Quality of Life Questionnaire, *SLEC* Stressful Life Events Checklist

Socioeconomic status measures of education and income were representative of the Canadian population [34]. Of the 72 (88%) reporting household income, 40.3% reported income equal or higher than the $100,000; 36.1% reported income of $51,000-100,000; 15.3% had 26,000-50,000: and 8.3% had less than 25,000 annually. Of the 70 (85.4%) reporting place of residence, 12.9% lived in rural areas (< 2000 people); 40% in small centers (2000 to 50,000), 7.1% in medium centers (> 50,000-100,000) and 37.2% in large population centers (> 100,000). Of the 75 (91%) fathers and 74 (90%) mothers reporting education, 74% of fathers and 78% of mothers reported at least some post-secondary education. Thirty-eight percent of fathers and 30% of mothers had university or post-graduate degrees.

At baseline, PAQ-C/A raw scores were lower in girls compared to boys (*p* = 0.026). Age and sex-matched PAQ-C/A z-scores were calculated for participants aged 8 to 15 years old and were significantly lower than the reference population (median (IQR) z-score − 0.356 (− 0.958–0.452); *p* = 0.005).

**Fig. 1:**
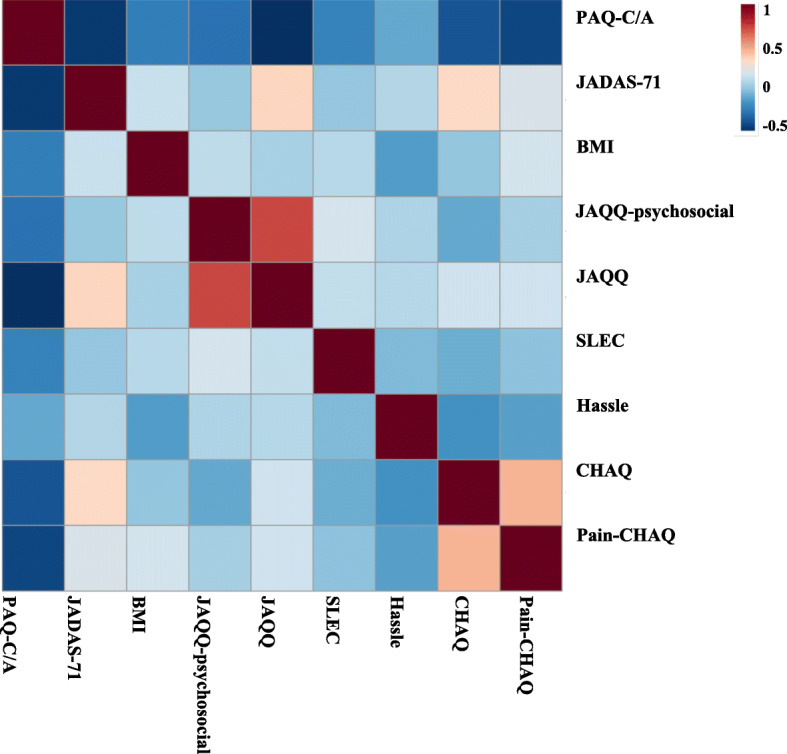
Correlation heat map at baseline. BMI, body mass index; PAQ-C/A score, physical activity questionnaire for children and adolescents; JADAS-71, Juvenile Arthritis Disease Activity Score; CHAQ, Child Health Assessment Questionnaire; JAQQ, Juvenile Arthritis Quality of Life Questionnaire; SLEC, Stressful Life Events Checklist

At baseline, disease activity (JADAS-71) and function (CHAQ) were negatively correlated with PA raw scores (*r* = − 0.496; *p* <  0.001 and *r* = − 0.291; *p* = 0.008, respectively). The CHAQ pain scale had a negative correlation with PA raw score at baseline (*r* = − 0.466; *p* < 0.001). The correlation with psychosocial stress was variable: the psychosocial domain of the JAQQ had a small negative correlation with PA raw score at baseline (*r* = − 0.251; *p* = 0.023), whereas the Hassles scale (*r* = 0.096, *p* = 0.389) and SLEC score (*r* = − 0.137, *p* = 0.216) showed no significant correlation. There was no significant relationship between PA raw score and socioeconomic status indicators. There were no differences in PA raw scores among JIA subtypes (data not shown). BMI at baseline was higher than the normative population for girls (BMI median percentile 63.6 (30.9–84.2, IQR)) but not boys (BMI median percentile 50.0 (30.0–86.5, IQR)). BMI at baseline was negatively correlated to PA (*r* = − 2.42, *p* = 0.029). Figure 1 shows a correlation heat map of these variables at baseline.

**Fig. 2:**
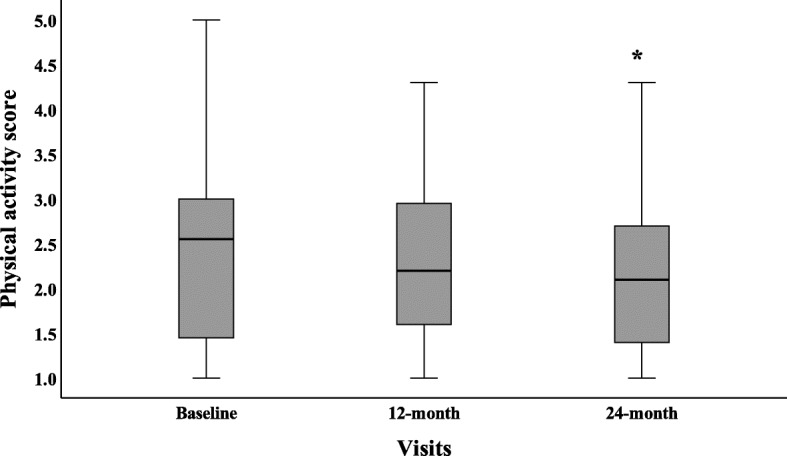
Boxplot of PAQ-C/A raw score at baseline, 12 and 24 months follow up. * There was a significant change in PAQ score from baseline to 24-month (*p* = 0.003)

There was a decline in PAQ-C/A raw scores over time, with a decline from baseline (median and IQR: 2.6, 1.4–3.1) to 12 months (median and IQR: 2.4, 1.7–3.0) and a further decline at 24 months (median and IQR: 2.1, 1.4–2.7; *p* = 0.003) (Fig. 2).

**Fig. 3:**
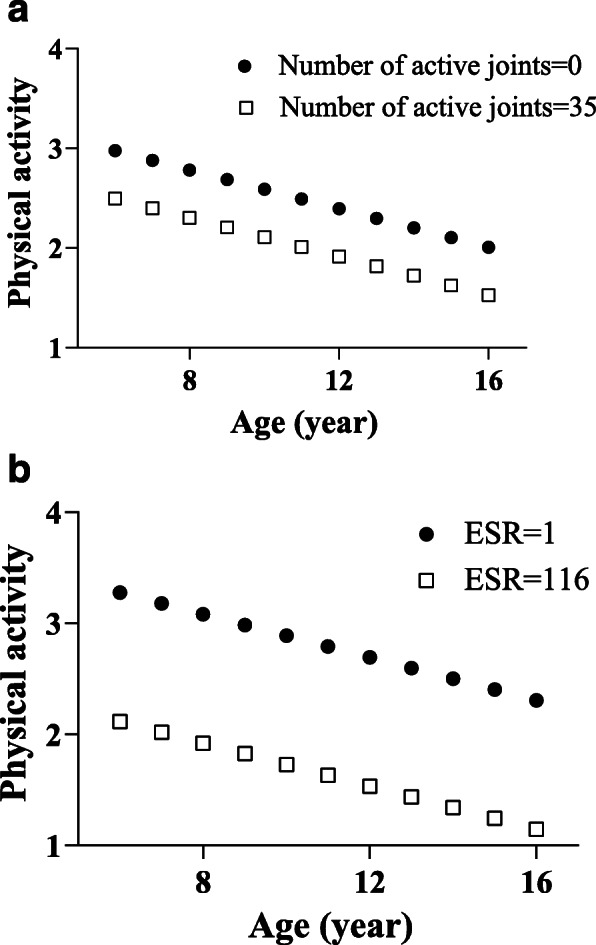
**a** Physical activity scores (PAQ-C/A) over time in children with low number of active joints (dark circle) and high number of active joints (open square). PAQ-C/A data is logarithmically transformed, fixed effect coefficient from LME model. **b** Physical activity scores (PAQC/A) over time in children with low ESR (dark circle) and high ESR (open square). PAQ-C/A data is logarithmically transformed, fixed effect coefficient from LME model

The model best fitted to our data was a linear mixed effect (LME) model evaluating the fixed effects of age, ESR, number of active joints and the random effect of the JAQQ-psychosocial domain and sex on the PAQ-C/A raw scores over a period of 2-years. As JADAS-71 is a composite score of ESR, CRP, PGA, and number of active joints, its relationship with PA was assessed separately. To apply the LME model, data were logarithmically transformed and auto scaled. Table 2 (LME results) shows that the PAQ-C/A raw scores in children with JIA decreased as age, disease duration, and ESR increased. The PA of the participants was also negatively influenced by an increase in disease activity as measured by the JADAS-71 (Table 3). The logarithmically transformed data and fixed effect coefficients in the LME model were used to create a figure illustrating that the decline in PAQ-C/A raw scores with age is greater for children with more active joints (Fig. 3a) and with higher ESR (Fig. 3b). Participants’ sex, CRP, CHAQ, Hassles scale, and SLEC score showed no significant association and were removed from the model.

**Table 2:** Fixed effects coefficients (95% CIs) describing effect on PAQ-C/A score calculated when random effect of JAQQ-psychosocial domain and sex were introduced to the model

Variables (fixed effects)	Estimates	*p*-value	95% CIs	
Intercept	0.188	**0.019**	0.030	0.345
Number of active joints	−0.094	**0.023**	−0.176	− 0.012
ESR	−0.150	**0.001**	−0.224	− 0.076
Age	−0.772	**0.001**	−0.846	− 0.697
12-month visit	−0.237	**0.016**	−0.430	− 0.044
24-month visit	−0.295	**0.002**	−0.486	− 0.104
lme_hierarchial = fitlme (PA,... [‘PA ~ 1 + Visits+ESR+ Number of active joints +Age+’... ‘(1|JAQQ_ psychosocial) + (1|Sex)’],'FitMethod’,'REML’)				

*CI* confidence interval, *ESR* erythrocyte sedimentation rate

The table shows that the fixed effect and random effect terms significantly affect the response (PAC-C/A)

**Table 3:** Fixed effects coefficients (95% CIs) describing effect on PAQ-C/A score calculated when random effect of JAQQ-

Variables (fixed effects)	Estimates	*p*-value	95% CIs	
Intercept	0.187	**0.031**	0.016	0.358
JADAS-71	−0.149	**< 0.001**	−0.229	− 0.069
Age	−0.761	**0.001**	−0.838	− 0.684
12-month visit	−0.202	**0.038**	−0.394	− 0.011
24-month visit	−0.305	**0.001**	−0.394	− 0.011
lme_hierarchial = fitlme (PA,... [PA ~ 1 + Visits+JADAS-71 + Age+’... ‘(1|JAQQ_ psychosocial) + (1|Sex)’],'FitMethod’,'REML’)				

*CI* confidence interval, *JADAS* Juvenile Arthritis Disease Activity Score

The table shows that the fixed effect and random effect terms significantly affect the response (PAC-C/A)

## Discussion

We address current gaps in knowledge on the trajectory of PA in JIA and its determinants and extend the current literature with three important observations. First, our prospective longitudinal cohort of children aged 6 to 16 years with JIA indicates PA levels decline over the first 2 years of disease despite declines in clinical disease activity. Second, higher levels of disease activity are associated with lower PA levels over the first 2 years of disease. Third, disease-specific psychosocial stress as measured by the JAQQ psychosocial domain has a negative association with PA, but we could not find associations with generic measures of psychosocial function, daily hassles and stressful life events, with PA. An effect of socioeconomic factors was not found.

### Physical activity and disease activity

We found that Canadian children with new onset JIA and moderate disease activity, pain and functional impairments have lower PA levels than healthy Canadian children [17, 33]. Our findings differ from recent reports that children with new onset JIA and low disease activity have similar PA levels to healthy children [2].

Consistent with the literature in healthy children, PA levels in our study were lower in girls and declined with age [1, 35]. We did not find differences in PA levels among JIA subtypes, but this could be explained by small patient numbers within certain subtypes. Previous studies have shown lower PA in rheumatoid factor positive polyarticular JIA [5] and enthesitis related arthritis [36]. It is notable that in our study the majority of children had polyarticular JIA, due to designed enrollment bias during the first 6 months of the study (only patients with polyarthritis and systemic arthritis were recruited during the first 6 months) and the exclusion of children younger than age 6 (oligoarthritis commonly presents at a younger age). This may partly explain the lower levels of PA in our cohort. It is also possible that our reference population PA levels (1991–1997) are not directly comparable. Children are reported to engage in less active commuting, high school physical education, and outdoor play but more organized sport than previous generations [37]. However despite changes in how children are active, long-term trends in PA over time appear to be stable. A multi-national study of trends in PA from 2002 to 2010 across 32 countries from Europe and North America reported a slight increase in 11-, 13- and 15-year-olds meeting the 60 min of daily PA recommendation (17.0% in 2002 and 18.6% in 2010) [38].

We found a negative correlation between PA and disease activity (JADAS-71), and this correlation increased from baseline to 12-month and 24-month follow-up, despite decreasing disease activity over time. These findings are in keeping with the existing literature which reports a negative relationship between PA and disease activity [5] as well as lower PA in JIA populations with good disease control [5, 6].

PA levels in our cohort progressively declined following diagnosis, with lower PA at 12 months and further decline in PA at 24 months. This pattern of declining PA with age is consistent with healthy children. Our results can be interpreted in the context of the large body of literature which reports lower levels of PA in children with established JIA [4–6]. Our longitudinal models show that in addition to age-related declines in PA, lower PA in children with JIA is associated with disease duration and disease activity, as measured by the JADAS-71, ESR and active joint counts. However, changes in disease activity do not solely explain the decline in PA. In keeping with our results, Norgaard and colleagues reported that disease-related factors account for a low percentage of the variance in PA levels of children with JIA [5]. As such, non-disease related factors must play a large role in the observed decline in PA during the first 2 years after diagnosis. A new diagnosis of JIA may lead to a change in family, peer and community supports’ (teachers, coaches) attitudes towards PA and sport participation. Concerns about disease flares, increased risk of injury or underperformance may limit a child’s PA. A recent study from Denmark found children with JIA had less participation in sport and less consistent participation in school-based physical education activity. Ninety percent (*N* = 62) reported pain in joints (81%) and/or muscles (30%) but non-arthritis related factors including shortness of breath/side stitches (30%), lack of competency in specific activities (30%), and lack of support from teachers (25%) were also reported as reasons for decreased school PA participation [39].

### Physical activity and psychosocial stress

Our study is the first to explore the joint relationship of psychosocial stress and SES with PA in children with JIA. Consistent with our hypothesis, higher levels of disease-specific psychosocial stress were associated with lower PA but this was apparent only with the disease-specific JAQQ psychosocial domain, and not with generic measures, and only in univariate tests, not in LME. Although the literature consistently reports that stress has a negative effect on PA in healthy populations [40], our patients apparently distinguished between arthritis-related and non-arthritis-related psychosocial stress. Alternatively, the arthritis-specific instrument was more sensitive; but in either case, our observation underscores the importance of using disease-specific measures. The lack of independent effects of the JAQQ psychosocial in LME may be due to possible interactions among variables or an inadequate sample size.

Personal factors of resilience, self-efficacy and support may mitigate the negative effects of stress and may explain the modest correlation relating PA to the JAQQ psychosocial scale as well as the lack of effect of generic stresses. Studies in healthy children and adolescents and those with disabilities, including JIA, have shown that parental support is positively correlated to PA [41]. In addition, Seid and colleagues reported that parental emotional distress, social support and child self-efficacy are related to changes in health-related quality of life and physical function in newly diagnosed children with JIA [13]. Children and parents who are habitually active as a family may increase or maintain their PA to cope with stress while families that are less habitually active may decrease their PA during stress. We did not capture parent PA level in our study and cannot test this hypothesis.

Our inability to relate SES to PA in children with JIA in our study may be due to small patient numbers in the face of the multiple categories within SES variables. Further studies with larger populations are required to clarify the effect of SES.

Our results highlight that PA is a complex behavior influenced by many factors. Family and child stress may affect PA level, either positively or negatively. The influence of psychosocial stress and the role of resiliency, motivation and self-efficacy in facilitating PA in children with JIA require further study.

### Study strengths and limitations

The strengths of our study include prospective longitudinal evaluations, using specific longitudinal trajectory models, of PA levels and the relationship of PA to disease activity and psychosocial stress; and the inclusion of both generic and disease-specific instruments. We acknowledge several limitations of our study. JIA is a heterogeneous disease and our cohort did not include children younger than age 6 (because measurement tools for PA and stress were not available for younger children). Our cohort is also skewed towards more severe disease (as a consequence of our recruitment protocol) and therefore is not representative of a usual JIA clinic population. Thus, our results may not be applicable to younger children and all subtypes of JIA. It may be difficult to compare our results to other studies, however, our goal was to generate novel information about PA levels and JIA category, disease activity and measures of SES and psychosocial stress. We used self-report measures of psychosocial stress and PA. While self-report measures have inherent bias, all the measures reported are commonly cited in pediatric research. We did not capture PA intensity or absolute time spent engaged in PA and are thus unable to comment on whether this cohort of children is meeting current PA recommendations of 1 h of moderate vigorous PA per day [42]. We did not measure resilience, motivation or self-efficacy, all of which may influence the effect of stress on PA. These will be important to consider in future studies. We did not capture medication side effects or fatigue, both of which may affect PA. The majority of our patients had moderate to high SES and we were unable to relate SES to PA. Further studies with larger populations and measurements of multiple PA domains (leisure, transportation, occupation, and housing) are needed to clarify the effects of SES. Finally, small patient numbers may be responsible for apparent lack of effects of certain variables.

## Conclusions

On average, Canadian children with newly diagnosed JIA had lower PA levels than healthy Canadian children. Furthermore, PA levels in our cohort decreased following diagnosis and were progressively lower at 12 and 24 months. The consistent decline in disease activity, the inverse relation of PA with disease activity, and its negative correlation with a psychosocial scale support an effect of psychosocial stress in impeding PA in children with JIA. Our observations also point to the importance of disease-specific measures. Our study demonstrates a clear need to better understand the relationship between PA, disease activity, SES, psychosocial stress, and factors mitigating stress in children with JIA. Future studies should focus on better understanding how health related quality of life, SES and patient reported outcomes (pain, stress, fatigue, efficacy, attitudes, barriers, motivation, social support,) relate to PA. Identifying those factors associated with increased PA may help healthcare providers identify strategies for PA promotion. Presently, promotion of age appropriate physical activity and safe sport participation remains an important part of JIA management.

## Supplementary Information

**Additional file 1.**

## Data Availability

The datasets used during the current study are available from the corresponding author on reasonable request.

## Abbreviations

BBOP: Biologically-based Outcome Predictors in JIA
BMI: Body mass index
CHAQ: Child Health Assessment Questionnaire
CRP: c-reactive protein
ESR: Erythrocyte sedimentation rate
ILAR: International League of Associations for Rheumatology
IQR: Interquartile range
JADAS-71: Juvenile Arthritis Disease Activity Score
JAQQ: Juvenile Arthritis Quality of Life Questionnaire
JIA: Juvenile idiopathic arthritis
LME: Linear mixed effect
PA: Physical activity
PAQ-A: Physical Activity Questionnaire for Adolescents
PAQ-C: Physical Activity Questionnaire for Children
PGA: Physician global assessment
RF: Rheumatoid factor
SES: Socioeconomic status
SLEC: Stressful life events checklist
VAS: Visual analog scale
