# Supplementary material for: Clinical and psychosocial stress factors are associated with decline in physical activity over time in children with juvenile idiopathic arthritis
Source: Pediatr Rheumatol Online J. 2021 Jun 29;19:97. doi: 10.1186/s12969-021-00584-4 (PMC8243495; doi:10.1186/s12969-021-00584-4)
Supplement: Supplementary file 1 — Additional file 1. [file 12969_2021_584_MOESM1_ESM.docx]

| Psychosocial Stress Questionnaire | Item examples |
| --- | --- |
| Juvenile Arthritis Quality of Life Questionnaire (JAQQ)  Psychosocial Function (as a result of arthritis or its treatment) | Got teased a lot.  Felt frustrated.  Felt worthless of inferior.  Demanded a lot of attention. |
| Children’s Hassles Scale | Kids at school teased you.  Your schoolwork was too hard.  Your mother or father didn’t have enough time to do something with you.  You got punished when you did something wrong. |
| Stressful Life Events Checklist (SLEC)  Child (C) - age less than 12 years  Adolescent (A) - age 12 years and older | Did you change schools? (C)  Did you change schools/colleges? (A)  Has a parent been unemployed? (C/A)  Were you hospitalized or did you have surgery? (C/A)  Were you a victim of any crimes? (C/A)  Has anyone in your immediate family passed away? (C/A) |

Supplementary Table 1. Examples of items on the Psychosocial Stress Questionnaires.


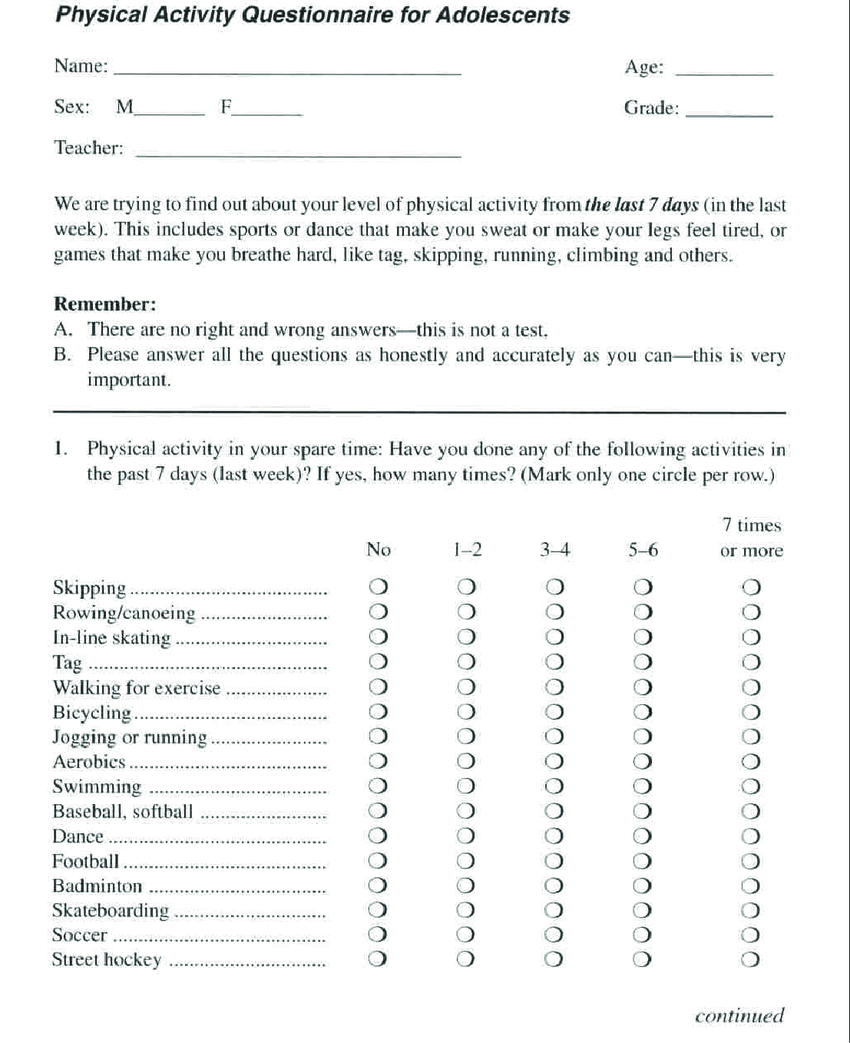


Supplementary Figure 1. First page of the Physical Activity Questionnaire for Adolescents
